# Supplementary material for: Examining the Impact of the COVID-19 Pandemic on Suicide-Attempt Survivors
Source: Int J Environ Res Public Health. 2025 Jul 4;22(7):1072. doi: 10.3390/ijerph22071072 (PMC12294532; doi:10.3390/ijerph22071072)
Supplement: Supplementary file 1 [file ijerph-22-01072-s001.zip › ijerph-3553903-supplementary.pdf]

Supplementary Materials

COVID-19 Impact on Functioning Scale

Due to COVID-19, do you feel like:	Not at all (1)	A little (2)	Somewhat (3)	Moderately (4)	Very much (5)	Completely (6)
1. You live in constant fear?						
2. You spend a lot of effort trying to stay mentally healthy?						
3. Your mental health has improved?						
4. Your mental health has suffered?						
5. You spend a lot of effort trying to stay physically healthy?						
6. Your physical health has improved?						
7. Your physical health has suffered?						
8. You have had financial challenges?						
9. You have enjoyed the extra time alone?						
10. You have enjoyed the extra time with family/people whom you live?						

Note: Items 3, 6, 9, and 10 are reverse coded.

Feelings of Despair Due to Pandemic Scale

After thinking about the coronavirus in the last few weeks:				
	Not at all (1)	Occasionally (2)	Some days (3)	Nearly every day over the last 2 weeks (4)
1. I felt extremely hopeless about the future.				
2. I felt extremely helpless, like I cannot do anything that will make things better.				
3. I wished I was already dead so I did not have to deal with the coronavirus.				
4. I wondered if God was angry with or had abandoned some people.				
5. I wondered if the leaders of our country brought this on.				
